# Non-Hodgkin’s lymphoma presenting with lower extremity edema and radiculopathy in a post-kidney transplant patient: A case report

**DOI:** 10.1097/MD.0000000000042943

**Published:** 2025-06-27

**Authors:** Jaesuk Kim, So Young Kwon

**Affiliations:** a Department of Anesthesiology and Pain Medicine, The Catholic University of Korea, St. Vincent’s Hospital, Suwon, Korea.

**Keywords:** edema, kidney transplantation, lymphoma, non-Hodgkin, radiculopathy

## Abstract

**Rationale:**

Non-Hodgkin lymphoma (NHL) can present with vague and nonspecific symptoms, making early diagnosis challenging, especially in immunocompromised patients. Kidney transplant recipients, due to long-term immunosuppressive therapy, have an increased risk of developing malignancies, including NHL.

**Patient concerns:**

A post-kidney transplantation patient presented with lower extremity symptoms. Initial lumbar spine magnetic resonance imaging suggested foraminal stenosis and disc herniation. However, persistent symptoms and lack of clinical improvement prompted further investigation. A contrast-enhanced computed tomography revealed a retroperitoneal mass compressing the iliac vessels. Biopsy confirmed diffuse large B-cell lymphoma.

**Diagnoses:**

Abdominal computed tomography showed multiple hepatic nodules, ascites, and right pleural effusion, consistent with systemic involvement of NHL.

**Interventions:**

The patient was treated with R-CHOP chemotherapy, the standard regimen for diffuse large B-cell lymphoma.

**Outcomes:**

The patient demonstrated a favorable response to chemotherapy, with symptomatic improvement and a reduction in the retroperitoneal mass size. Continued follow-up emphasized supportive management and oncologic surveillance.

**Lessons:**

This case highlights the need for a broad differential diagnosis in posttransplant patients with common musculoskeletal complaints. Early consideration of malignancy is crucial to avoid delayed diagnosis and optimize outcomes.

## 1. Introduction

Lower extremity edema is a common clinical symptom with a broad differential diagnosis, ranging from benign causes such as venous insufficiency to more serious conditions of deep vein thrombosis or malignancy.^[1,2]^ In patients who have undergone kidney transplantation (KT), the long-term use of immunosuppressive therapy significantly increases the risk of malignancies, particularly non-Hodgkin lymphoma (NHL).^[3–5]^ NHL can present with a variety of symptoms, often complicating timely diagnosis, especially when the symptoms mimic more common conditions like spinal pathologies.^[6]^

This case report describes a post-KT patient who developed significant lower extremity edema, leading to the diagnosis of diffuse large B-cell lymphoma (DLBCL). The patient had been followed closely for many years after KT, but the new-onset edema and subsequent investigation revealed an underlying malignancy. This case underscores the importance of considering a broad differential diagnosis in posttransplant patients presenting with new or atypical symptoms, even long after the transplant.

## 2. Case presentation

The reporting of this study conforms to CARE guidelines.

A 77-year-old female with a significant past medical history, including hypertension, angina, and a KT performed 15 years prior, presented to the pain clinic with new-onset left thigh numbness and lower extremity pain. The patient had been on long-term immunosuppressive therapy since the KT. Her past surgical history included bilateral nephrectomy, splenectomy, and multiple other procedures, with regular follow-up visits to monitor her posttransplant status.

**Figure 1.**
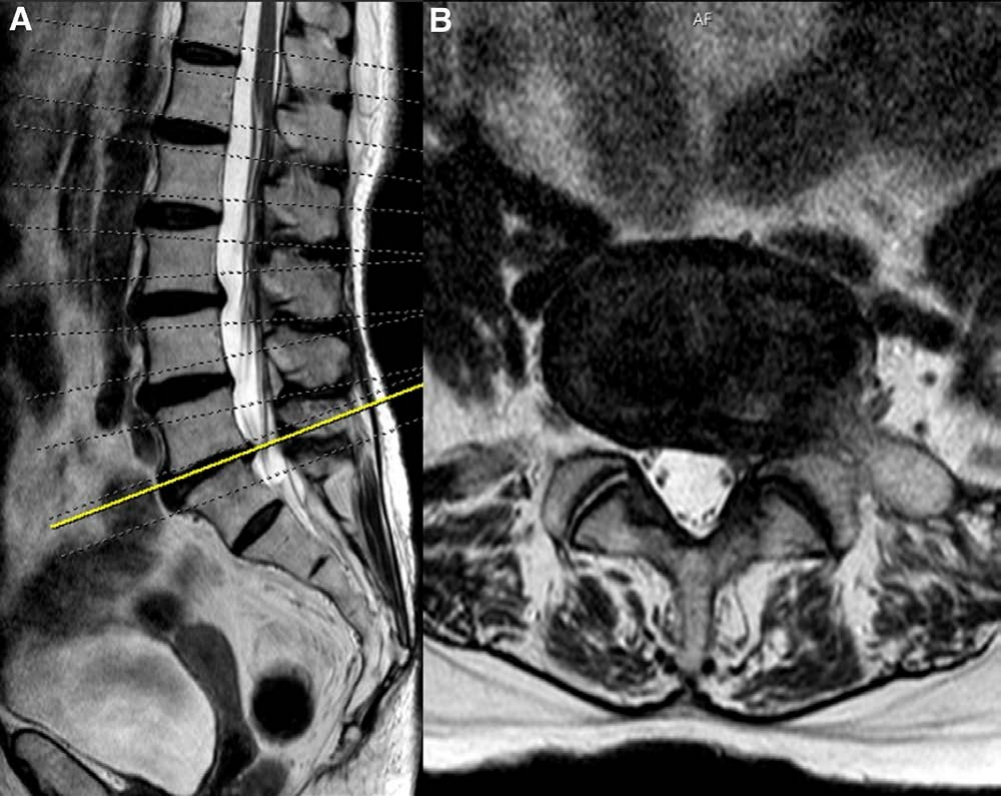
Lumbar spine MRI showing lumbar 5-sacrum 1 foraminal stenosis and disc extrusion ((A) sagittal view and (B) axial view). MRI = magnetic resonance imaging.

Several months prior to the current evaluation, the patient experienced a tingling sensation in her left thigh. Magnetic resonance imaging of the lumbar spine was conducted, revealing findings of lumbarization at L4–5, right foraminal herniation at L5–6, and left foraminal stenosis (Fig. 1). Based on these imaging results, her symptoms were initially attributed to lumbar spine pathology. She was treated with a buprenorphine transdermal patch, which provided temporary relief.

**Figure 2.**
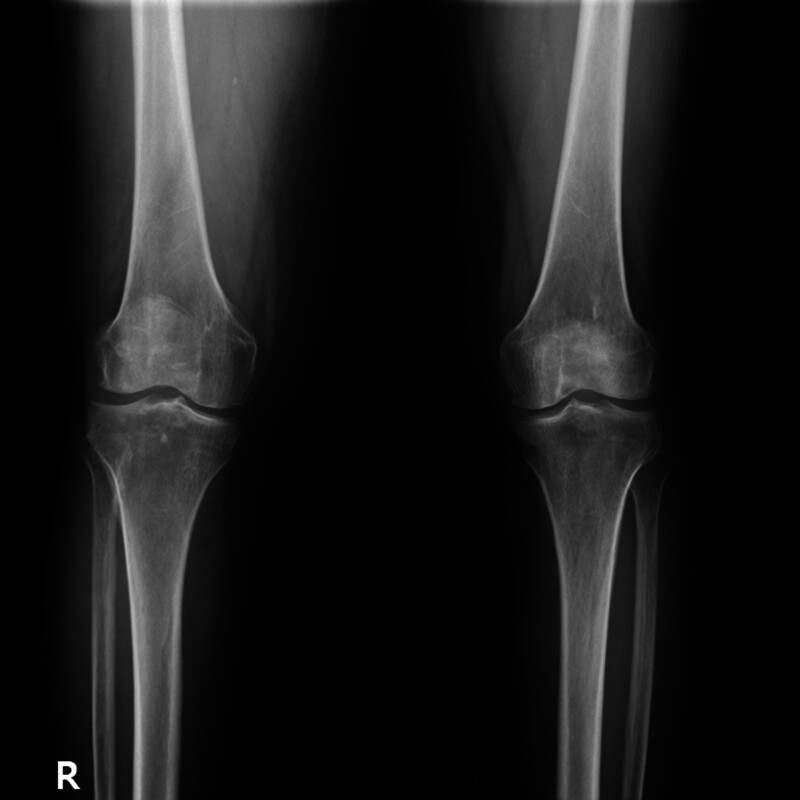
Knee X-ray in AP view, showing bilateral osteoarthritis (Kellgren–Lawrence grade II).

However, a few months later, the patient’s symptoms progressed, leading to significant pain and swelling in the right knee and lower leg. A knee radiograph was performed to reveal Kellgren–Lawrence grade II osteoarthritis, and an intra-articular steroid injection was administered (Fig. 2). While the patient’s pain partially improved, swelling of the right leg persisted and worsened, prompting further investigation.

**Figure 3.**
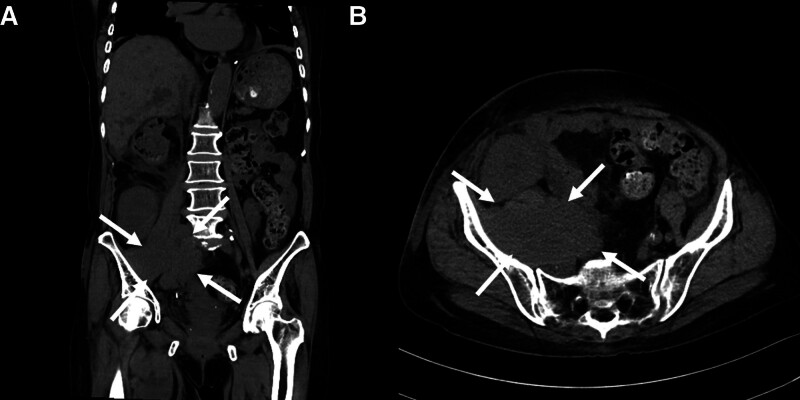
Contrast-enhanced CT scan revealing retroperitoneal mass compressing the iliac vessels ((A) coronal view and (B) axial view).

Shortly thereafter, the patient was referred to the emergency department due to concerns of vascular compromise. A contrast-enhanced CT scan of the lower extremities revealed a large heterogeneous mass, approximately 9.9 × 6.4 cm in size, located in the right retroperitoneum and iliopsoas muscle (Fig. 3). This mass was causing significant compression of the right iliac vessels, including obliteration of the right common, external, and internal veins, and encasement of the corresponding arteries. These findings raised suspicion for a malignant process.

Approximately 1 month after the onset of swelling, a laparoscopic lymph node excisional biopsy was performed. The biopsy confirmed the diagnosis of DLBCL, Germinal Center B-cell-like subtype, arising in immune deficiency/dysregulation setting (posttransplantation). Epstein–Barr virus was negative by immunohistochemistry but focally positive in scattered cells by in situ hybridization. Human herpes virus-8 was negative.

Staging was performed using PET-CT, which revealed a hypermetabolic mass involving the right iliac chain and iliopsoas muscle. Additional FDG uptake was noted in the left paraaortic space, left external iliac area, and right axilla. Chest and neck CT scans showed no evidence of lymphoma involvement. Based on these findings, the disease was classified as Stage III (Ann Arbor classification).

Following the diagnosis, the patient commenced treatment with R-CHOP chemotherapy. The initial immunosuppressive regimen included Tacrolimus (1.5 mg/day) and Deflazacort (3 mg/day). After lymphoma diagnosis, the regimen was adjusted to Tacrolimus (1 mg/day), Deflazacort (36 mg/day), and Everolimus (3 mg/day). Following completion of R-CHOP chemotherapy, immunosuppressive therapy was readjusted to Tacrolimus (1 mg/day), Deflazacort (6 mg/day), and Everolimus (2 mg/day). The patient remained continuously on immunosuppression throughout the course.

The diagnosis of lymphoma, which had initially been masked by symptoms mimicking lumbar radiculopathy, underscores the complexity of managing long-term post-KT patients and highlights the importance of considering malignancy in differential diagnoses, even many years after transplantation.

## 3. Discussion

This case highlights not only the diagnostic complexity associated with lower extremity edema and radiculopathy in a post-KT patient, but also the potential pitfalls of focusing too narrowly on the presumed diagnosis.

Lower extremity edema can arise from a wide range of conditions, including benign causes like venous insufficiency or lymphedema, as well as more serious causes such as deep vein thrombosis or, as in this case, malignancy.^[1,2]^ In a patient with a history of long-term immunosuppression post-KT, the differential diagnosis must remain broad, with a high index of suspicion for malignancies such as NHL.^[3]^

Initially, the patient’s symptoms of left thigh numbness and radiculopathy led to an magnetic resonance imaging of the lumbar spine, which revealed findings typical of foraminal stenosis and herniation. These findings appeared to explain the patient’s symptoms, and treatment was administered accordingly. However, the sudden onset of significant right lower extremity edema prompted further investigation, leading to the discovery of a large retroperitoneal mass compressing the iliac vessels. The rapid progression of edema and its persistence despite initial conservative treatment were key signs of something more serious.^[7]^

NHL, especially in posttransplant patients, can manifest with a wide variety of symptoms depending on the site of involvement.^[6]^ In this patient, the lymphoma presented with localized compression of the iliac vessels, causing significant edema and radiculopathy-like symptoms in the lower extremity. This case demonstrates that, in immunosuppressed patients, even relatively common symptoms like edema can be indicative of underlying malignancies.^[8,9]^

Importantly, the discovery of the lymphoma occurred 15 years posttransplant, emphasizing the need for ongoing vigilance in the long-term follow-up of post-KT patients. Even many years after transplantation, new-onset symptoms such as unexplained edema or neurological changes should prompt a thorough diagnostic evaluation, including imaging and biopsy when necessary. The overlap of clinical features between benign and malignant conditions, as seen in this case, can lead to diagnostic delays if malignancy is not considered early in the differential diagnosis.

This case also serves as a reminder of the importance of comprehensive imaging and timely biopsy in the diagnostic process. In this patient, the discovery of the retroperitoneal mass was crucial in redirecting the clinical approach and ensuring the appropriate treatment was initiated.

## 4. Conclusion

This case report highlights the complexity of diagnosing lower extremity radiculopathy in a post-kidney transplant patient, where the underlying cause was a malignant lymphoma. It underscores the need for clinicians to maintain a high index of suspicion for malignancies in posttransplant patients presenting with atypical or evolving symptoms, even many years after the transplant. The overlap of clinical features between benign and malignant conditions, as seen in this case, can lead to diagnostic delays. Therefore, thorough evaluation and consideration of alternative diagnoses are essential, particularly when initial treatments do not achieve the desired results. Early recognition and intervention are critical in improving patient outcomes, as demonstrated by the initiation of R-CHOP chemotherapy following the correct diagnosis of DLBCL.

## Author contributions

**Conceptualization:** So Young Kwon.

**Resources:** So Young Kwon.

**Writing – original draft:** Jaesuk Kim.

**Writing – review & editing:** Jaesuk Kim, So Young Kwon.
